# Supplementary material for: Analysis of time-to-positivity data in tuberculosis treatment studies: Identifying a new limit of quantification
Source: medRxiv. 2024 May 7:2024.05.06.24306879. Preprint. [Version 1] doi: 10.1101/2024.05.06.24306879 (PMC11100935; doi:10.1101/2024.05.06.24306879)
Supplement: Supplement 1 [file media-1.pdf]

Analysis of time-to-positivity data in tuberculosis treatment studies: Identifying a new limit of quantification

Suzanne M. Dufault[a,b#],Geraint R. Davies[c],Elin M. Svensson[d,e],Derek J. Sloan[f], Andrew D. McCallum[g], Anu Patel[h], Pieter Van Brantegem[i], Paulo Denti[j], Patrick P. J. Phillips[b,h]

a Division of Biostatistics, University of California, San Francisco, San Francisco, California, USA
b UCSF Center for Tuberculosis, University of California, San Francisco, San Francisco, California, USA
c Institute of Infection and Global Health, University of Liverpool, Liverpool, UK
d Department of Pharmacy, Radboud University Medical Center, Nijmegen, The Netherlands
e Department of Pharmacy, Uppsala University, Uppsala, Sweden
f School of Medicine, University of St Andrews, St Andrews, United Kingdom
g Department of Infectious Diseases, Oxford University Hospitals NHS Foundation Trust, Oxford, UK
h Division of Pulmonary and Critical Care Medicine, University of California, San Francisco, California, USA
i Department of Bioengineering and Therapeutic Sciences, University of California, San Francisco, California, USA
j Division of Clinical Pharmacology, Department of Medicine, University of Cape Town, South Africa

Supplemental Material

S1.1 Proportion of all TTP samples in [25,42] range

Table S1. The proportion of all sputum samples with TTP values in the range [25,42) for sputum samples collected from baseline to eight weeks post-randomization.

Trial	No. Samples	Proportion of Samples
REMoxTB	520	0.035
PanACEA MAMS-TB	218	0.071
NC-002 (PaMZ)	140	0.046
NC-005 (BPaMZ)	174	0.048
NC-006 (STAND, PaMZ)	362	0.079
Study 29	137	0.052
Study 29X	128	0.071

S1.2 Distribution of TTP samples by week

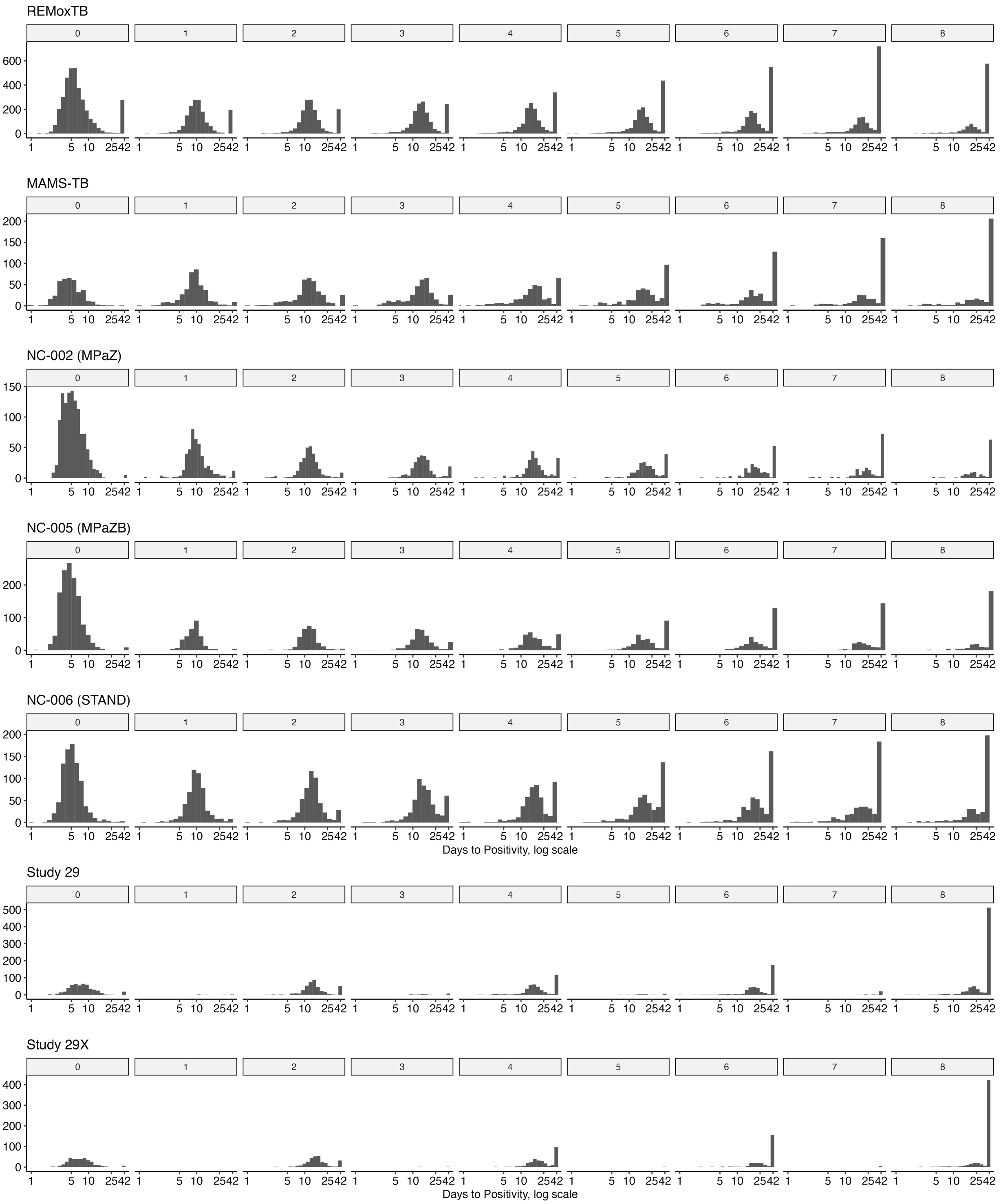

Fig S1. Histograms showing the distribution of TTP values for each week's sample. "Negative" values are recorded as 42 days.

S1.3 Bayesian Model Specification

This section aims to follow best practices in reporting Bayesian analyses by following the Bayesian Analysis Reporting Guidelines [17].

S1.3.1 Data variables

The dependent variable (y_{ijk}) is the $\log_{10}$(TTP) measured from the sputum sample given by individual i on regimen j at visit k and is modeled as a function of time since randomization (in weeks) (t_{ijk}). Let Q_{ijk} be an indicator variable that takes the value 1 when $y_{ijk} \geq \log(\text{ULOQ}_M)$, and 0 otherwise. In other words, Q_{ijk} denotes when a value is above the limit of quantification and therefore cannot be included quantitatively in the model. Instead, these values are handled as "right-censored" [18]. The linear mixed effects model is specified as in Eq. 2, with "brms" default priors used for all parameters except γ_0.

$$
\begin{aligned}
y_{ijk} &\sim \begin{cases} N(\mu, \sigma_1) & Q_{ijk} = 0 \\ 1 - \Phi(\frac{\text{ULOQ}-\mu}{\sigma_1}) & Q_{ijk} = 1 \end{cases} \\
\mu &= \gamma_{0ij} + \gamma_{1ij} t_{ijk} \\
\sigma_1 &\sim \text{Student } t(3, 0, 2.5) \\
\gamma_{0ij} &\sim N(\gamma_0, s_1) \\
\gamma_{1ij} &\sim N(\gamma_1, s_2) \\
\gamma_0 &\sim N(0, 4^2) \\
\gamma_1 &\sim \text{Flat prior (“brms” default)} \\
s_1 &\sim \text{Student } t(3, 0, 2.5) \\
s_2 &\sim \text{Student } t(3, 0, 2.5)
\end{aligned}
\tag{2}
$$

S1.3.2 Code and software

All analyses were performed using the "brms" package in the R statistical software. All code used to perform these analyses is available at a GitHub repository managed by the first author (`https://github.com/sdufault15/ttp-lod`).

S1.3.3 Model results and fit

Model results are included in S2 at the regimen-level. Model statistics reflecting fit and convergence at the population-level are included in Tables S3 - S9. Figure S3 contains the posterior predictive checks for the main analysis models.

Table S2. Posterior point estimates (mode) and 95% highest-density credible intervals (HCI) for the regimen-level slopes from the linear models when the TTP diagnostic LOD and different $ULOQ_M$ thresholds are applied.

	Diagnostic LOD	$ULOQ_M$	
Regimen j	42 Day: $\hat{\gamma}_{1j}$ (95% HCI)	30 Day: $\hat{\gamma}_{1j}$ (95% HCI)	25 Day: $\hat{\gamma}_{1j}$ (95% HCI)
**PanACEA MAMS-TB**			
HR20ZM	0.138 (0.124, 0.154)	0.132 (0.117, 0.146)	0.127 (0.114, 0.142)
HR20ZQ	0.121 (0.104, 0.137)	0.116 (0.101, 0.130)	0.117 (0.098, 0.129)
HR35ZE	0.144 (0.126, 0.160)	0.138 (0.123, 0.155)	0.137 (0.121, 0.155)
HRZE	0.128 (0.116, 0.138)	0.122 (0.109, 0.131)	0.120 (0.109, 0.131)
HRZQ	0.122 (0.104, 0.137)	0.115 (0.098, 0.129)	0.115 (0.097, 0.128)
**REMoxTB**			
HRZE	0.095 (0.090, 0.100)	0.089 (0.085, 0.093)	0.086 (0.082, 0.090)
MHRZ	0.104 (0.100, 0.109)	0.097 (0.094, 0.102)	0.095 (0.091, 0.099)
EMRZ	0.107 (0.102, 0.111)	0.099 (0.095, 0.103)	0.097 (0.093, 0.100)
**NC-002 (PaMZ)**			
$Pa_{100}MZ$	0.154 (0.137, 0.172)	0.148 (0.131, 0.167)	0.149 (0.131, 0.166)
$Pa_{200}MZ$	0.144 (0.128, 0.158)	0.138 (0.123, 0.152)	0.137 (0.123, 0.152)
HRZE	0.126 (0.111, 0.146)	0.122 (0.109, 0.139)	0.125 (0.109, 0.141)
**NC-005 (BPaMZ)**			
HRZE	0.123 (0.111, 0.137)	0.118 (0.108, 0.130)	0.119 (0.107, 0.130)
$B_{200}PaZ$	0.143 (0.130, 0.156)	0.134 (0.123, 0.146)	0.132 (0.121, 0.144)
$B_{load}PaZ$	0.137 (0.127, 0.151)	0.131 (0.120, 0.142)	0.128 (0.117, 0.138)
**NC-006 (STAND, PaMZ)**			
HRZE	0.100 (0.090, 0.108)	0.094 (0.085, 0.103)	0.093 (0.085, 0.102)
$Pa_{100}MZ$	0.104 (0.097, 0.114)	0.099 (0.091, 0.107)	0.097 (0.089, 0.105)
$Pa_{200}MZ$	0.117 (0.107, 0.125)	0.109 (0.099, 0.118)	0.106 (0.096, 0.116)
$Pa_{200}MZ$	0.105 (0.097, 0.113)	0.099 (0.091, 0.107)	0.097 (0.088, 0.105)
**Study 29**			
$P_{10}HZE$	0.108 (0.101, 0.116)	0.095 (0.089, 0.101)	0.091 (0.084, 0.096)
HRZE	0.111 (0.105, 0.119)	0.099 (0.093, 0.105)	0.094 (0.088, 0.100)
**Study 29X**			
HRZE	0.122 (0.107, 0.138)	0.111 (0.097, 0.125)	0.110 (0.094, 0.123)
$P_{10}HZE$	0.134 (0.121, 0.147)	0.122 (0.111, 0.135)	0.121 (0.109, 0.133)
$P_{15}HZE$	0.133 (0.120, 0.148)	0.121 (0.109, 0.135)	0.120 (0.107, 0.133)
$P_{20}HZE$	0.141 (0.125, 0.156)	0.125 (0.114, 0.141)	0.126 (0.114, 0.143)

Table S3. REMoxTB model results.

Coefficient	Estimate	Est.Error	95% HCI	Rhat	Bulk ESS	Tail ESS
**$ULOQ_M$ = 25**						
γ_0	0.86	0.04	(0.81,0.92)	1.001	2540.6	1986.2
γ_1	0.09	0.02	(0.03,0.13)	1.006	550.3	246.8
σ_1	0.22	0.00	(0.21,0.22)	1.002	4622.1	4396.9
**$ULOQ_M$ = 30**						
γ_0	0.87	0.05	(0.79,0.93)	1.001	2668.6	1896.3
γ_1	0.10	0.02	(0.05,0.14)	1.003	1761.4	1335.9
σ_1	0.23	0.00	(0.23,0.24)	1.002	4476.4	3651.7
**LOD = 42**						
γ_0	0.87	0.08	(0.77,1.01)	1.021	314.1	253.6
γ_1	0.10	0.03	(0.06,0.16)	1.005	574.3	508.0
σ_1	0.25	0.00	(0.25,0.26)	1.005	974.4	1751.1

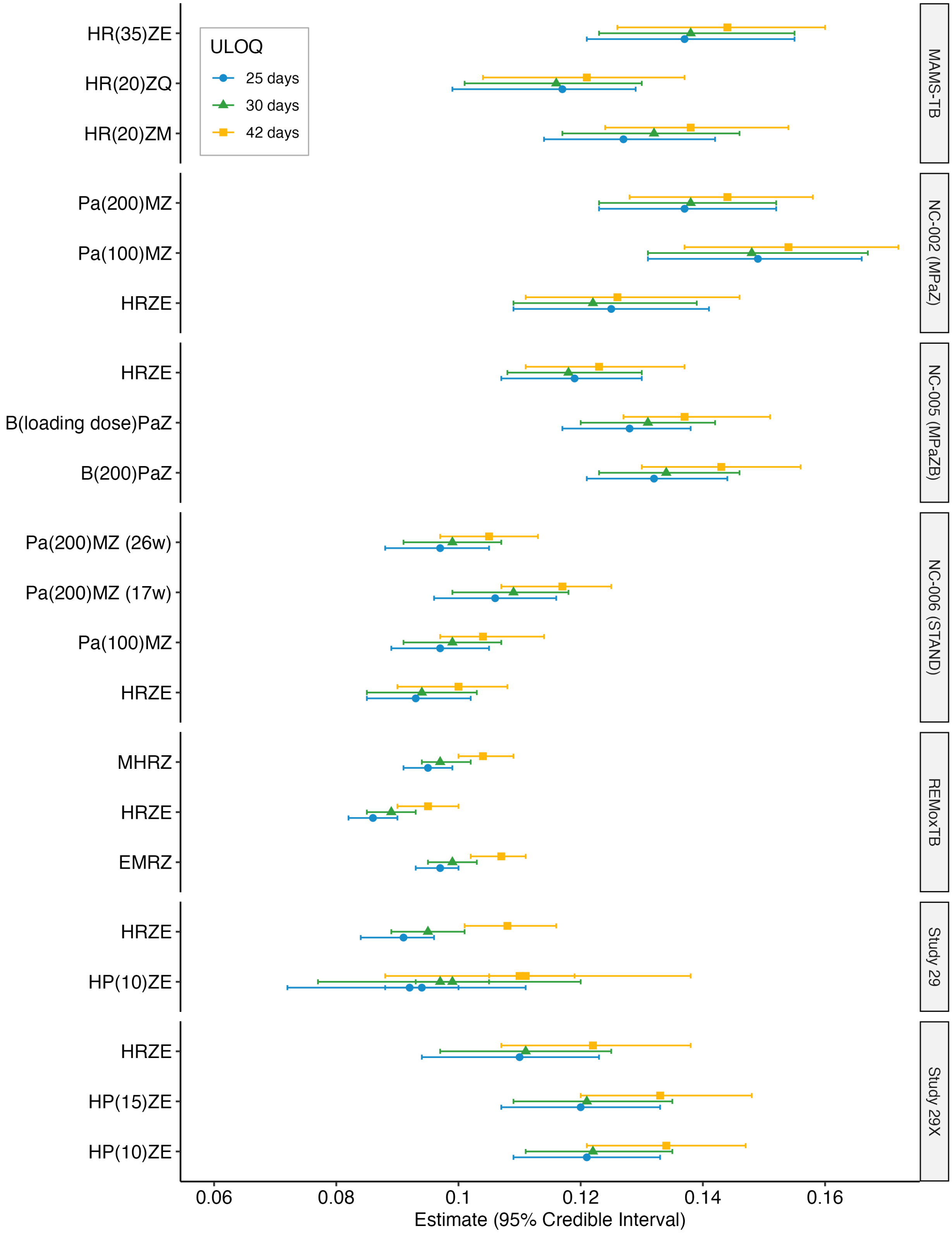

Fig S2. Forest plot of regimen-level estimates and 95% HCI

Table S4. PanACEA MAMS-TB model results.

Coefficient	Estimate	Est.Error	95% HCI	Rhat	Bulk ESS	TailESS
**$ULOQ_M$ = 25**						
γ_0	0.76	0.02	(0.73,0.80)	1.000	2954.7	2264.4
γ_1	0.12	0.01	(0.10,0.15)	1.005	1100.1	751.3
σ_1	0.26	0.00	(0.25,0.27)	1.000	2542.7	2291.0
**$ULOQ_M$ = 30**						
γ_0	0.76	0.02	(0.73,0.80)	1.000	3045.1	2320.7
γ_1	0.12	0.01	(0.10,0.15)	1.004	1433.4	1001.3
σ_1	0.26	0.00	(0.26,0.27)	1.002	2983.7	2721.8
**LOD = 42**						
γ_0	0.76	0.02	(0.72,0.80)	1.001	3390.1	2943.4
γ_1	0.13	0.01	(0.11,0.16)	1.002	1527.9	1009.2
σ_1	0.28	0.00	(0.27,0.28)	1.001	3168.5	2510.8

Table S5. NC-002 (PaMZ) model results.

Coefficient	Estimate	Est.Error	95% HCI	Rhat	Bulk ESS	Tail ESS
**$ULOQ_M$ = 25**						
γ_0	0.75	0.08	(0.62,0.90)	1.006	702.6	1011.4
γ_1	0.13	0.09	(-0.04,0.26)	1.028	179.9	123.0
σ_1	0.15	0.00	(0.14,0.15)	1.005	623.2	1262.0
**$ULOQ_M$ = 30**						
γ_0	0.75	0.08	(0.61,0.89)	1.003	752.5	887.7
γ_1	0.14	0.08	(0.04,0.33)	1.017	377.5	170.9
σ_1	0.15	0.00	(0.15,0.16)	1.002	1168.0	2327.6
**LOD = 42**						
γ_0	0.75	0.11	(0.57,0.92)	1.047	275.1	546.0
γ_1	0.14	0.06	(-0.01,0.25)	1.011	405.7	302.6
σ_1	0.16	0.00	(0.16,0.17)	1.010	289.8	584.2

Table S6. NC-005 (BPaMZ) model results.

Coefficient	Estimate	Est.Error	95% HCI	Rhat	Bulk ESS	Tail ESS
**$ULOQ_M$ = 25**						
γ_0	0.73	0.05	(0.66,0.82)	1.002	2053.8	2159.1
γ_1	0.13	0.04	(0.06,0.20)	1.007	982.6	723.5
σ_1	0.16	0.00	(0.16,0.16)	1.000	3552.5	4313.0
**$ULOQ_M$ = 30**						
γ_0	0.73	0.07	(0.65,0.84)	1.003	1030.1	1650.8
γ_1	0.13	0.04	(0.04,0.21)	1.004	726.2	523.9
σ_1	0.17	0.00	(0.16,0.17)	1.006	1368.0	2715.9
**LOD = 42**						
γ_0	0.72	0.07	(0.61,0.82)	1.003	1259.5	1976.4
γ_1	0.13	0.05	(0.03,0.23)	1.007	962.3	1082.0
σ_1	0.18	0.00	(0.17,0.18)	1.002	2516.9	3924.4

Table S7. NC-006 (STAND, PaMZ) model results.

Coefficient	Estimate	Est.Error	95% HCI	Rhat	Bulk ESS	Tail ESS
**ULOQ$_M$ = 25**						
γ_0	0.83	0.03	(0.79,0.89)	1.002	2943.0	2333.9
γ_1	0.09	0.01	(0.08,0.10)	1.001	2896.1	3108.1
σ_1	0.20	0.00	(0.20,0.21)	1.001	3053.1	5209.5
**ULOQ$_M$ = 30**						
γ_0	0.84	0.02	(0.78,0.89)	1.001	2769.6	3095.5
γ_1	0.08	0.01	(0.07,0.10)	1.002	2234.3	1782.1
σ_1	0.20	0.00	(0.20,0.21)	1.003	2629.9	3753.3
**LOD = 42**						
γ_0	0.84	0.03	(0.79,0.90)	1.004	1889.5	1674.5
γ_1	0.08	0.01	(0.07,0.09)	1.002	2354.7	2054.0
σ_1	0.20	0.00	(0.19,0.21)	1.006	1371.4	4309.1

Table S8. Study 29 model results.

Coefficient	Estimate	Est.Error	95% HCI	Rhat	Bulk ESS	Tail ESS
**ULOQ$_M$ = 25**						
γ_0	0.92	0.07	(0.80,1.05)	1.003	4367.8	3765.9
γ_1	0.09	0.02	(0.06,0.13)	1.002	2919.8	617.7
σ_1	0.20	0.00	(0.19,0.21)	1.001	2064.0	702.8
**ULOQ$_M$ = 30**						
γ_0	0.92	0.08	(0.77,1.08)	1.002	3536.7	2537.8
γ_1	0.10	0.02	(0.05,0.14)	1.006	2133.2	1573.3
σ_1	0.21	0.00	(0.20,0.22)	1.000	1764.5	3103.1
**LOD = 42**						
γ_0	0.92	0.14	(0.73,1.12)	1.001	2081.2	1029.8
γ_1	0.11	0.03	(0.07,0.16)	1.002	2727.5	1818.1
σ_1	0.23	0.01	(0.22,0.24)	1.001	1976.1	3644.8

Table S9. Study 29X model results.

Coefficient	Estimate	Est.Error	95% HCI	Rhat	Bulk ESS	Tail ESS
**ULOQ$_M$ = 25**						
γ_0	0.89	0.03	(0.85,0.94)	1.001	4674.3	4053.9
γ_1	0.12	0.02	(0.09,0.15)	1.005	1889.7	1803.0
σ_1	0.20	0.01	(0.19,0.21)	1.002	2117.6	4125.4
**ULOQ$_M$ = 30**						
γ_0	0.90	0.03	(0.85,0.95)	1.000	6261.9	5252.2
γ_1	0.12	0.01	(0.09,0.15)	1.002	2041.5	1515.1
σ_1	0.20	0.01	(0.19,0.21)	1.002	2375.2	4456.4
**LOD = 42**						
γ_0	0.89	0.03	(0.84,0.94)	1.001	5871.9	4704.7
γ_1	0.13	0.02	(0.10,0.16)	1.000	2352.5	1539.9
σ_1	0.22	0.01	(0.21,0.23)	1.001	2101.2	4780.5

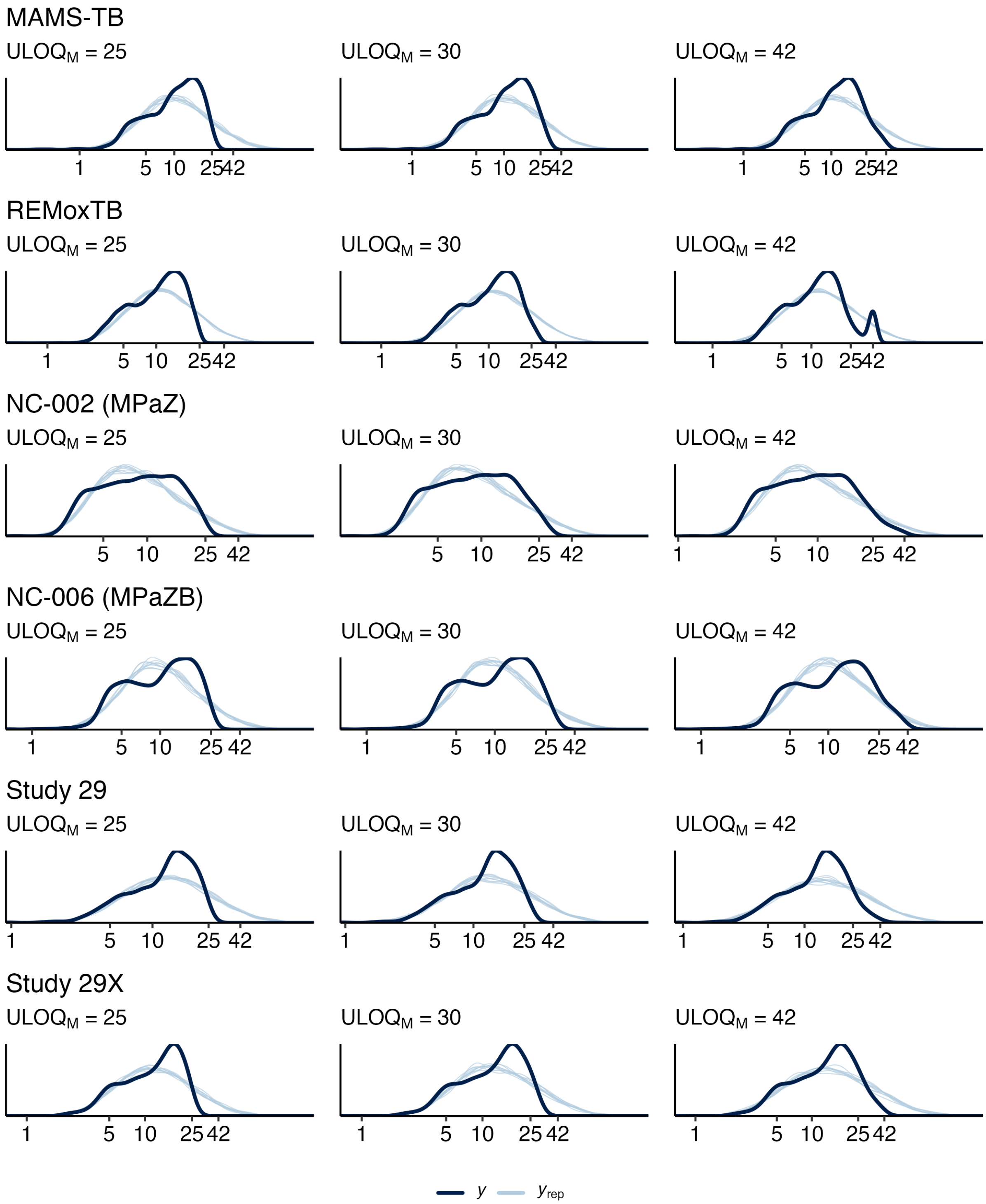

Note: Responses above the ULOQ are not shown.

Fig S3. Posterior predictive checks comparing the observed response data (y) to data drawn from the posterior of the model (y_{rep}).

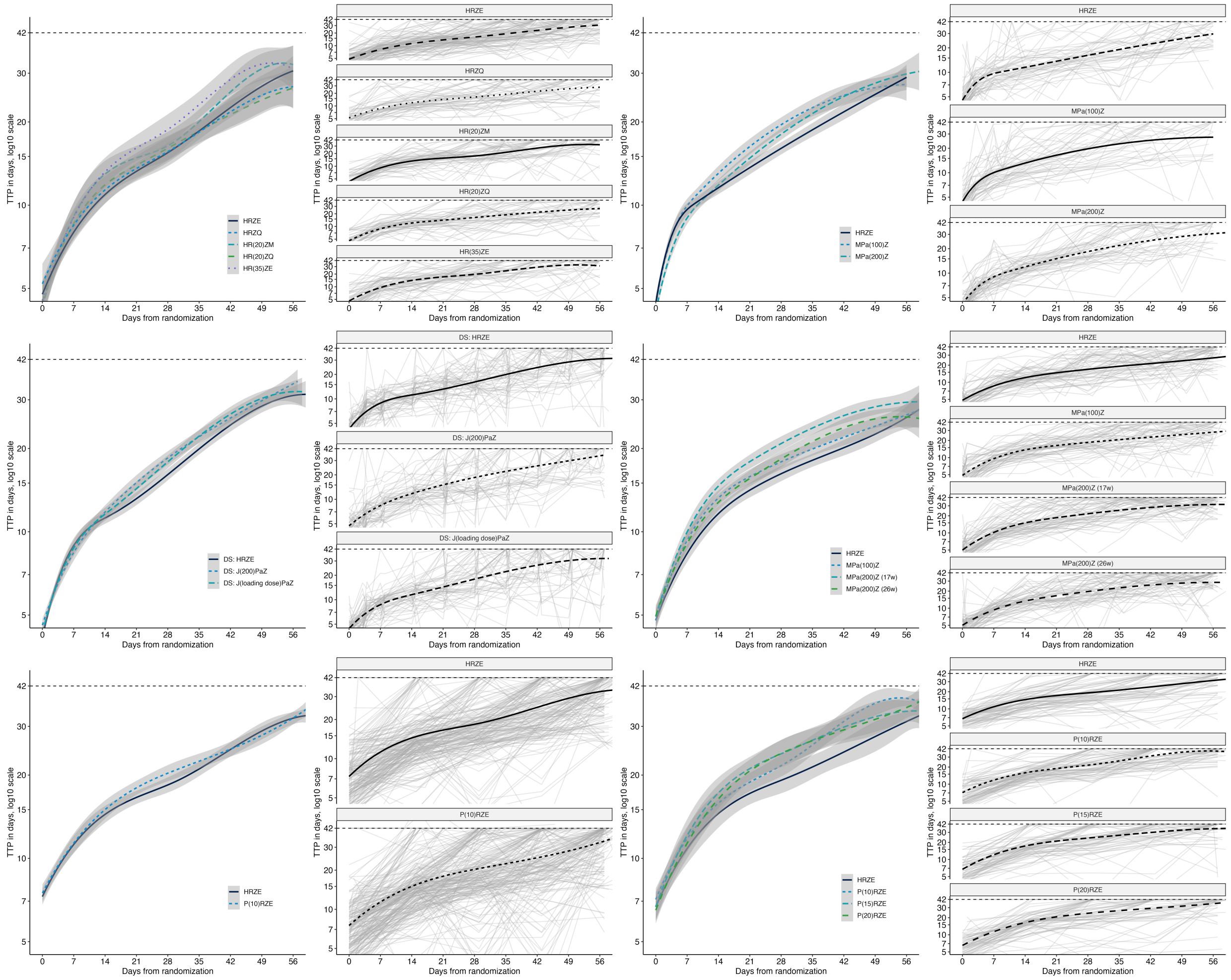

Fig S4. Observed time-to-positivity trajectories. Any observations at or above the diagnostic limit of detection (42 days) are recorded as 42 days. A: Regimen-level trends in TTP (lines) and estimated STAND, PaMZard errors (ribbons) as fit by smoothing splines. B: Individual TTP trajectories (light gray) and regimen-level smoothing spline (black).

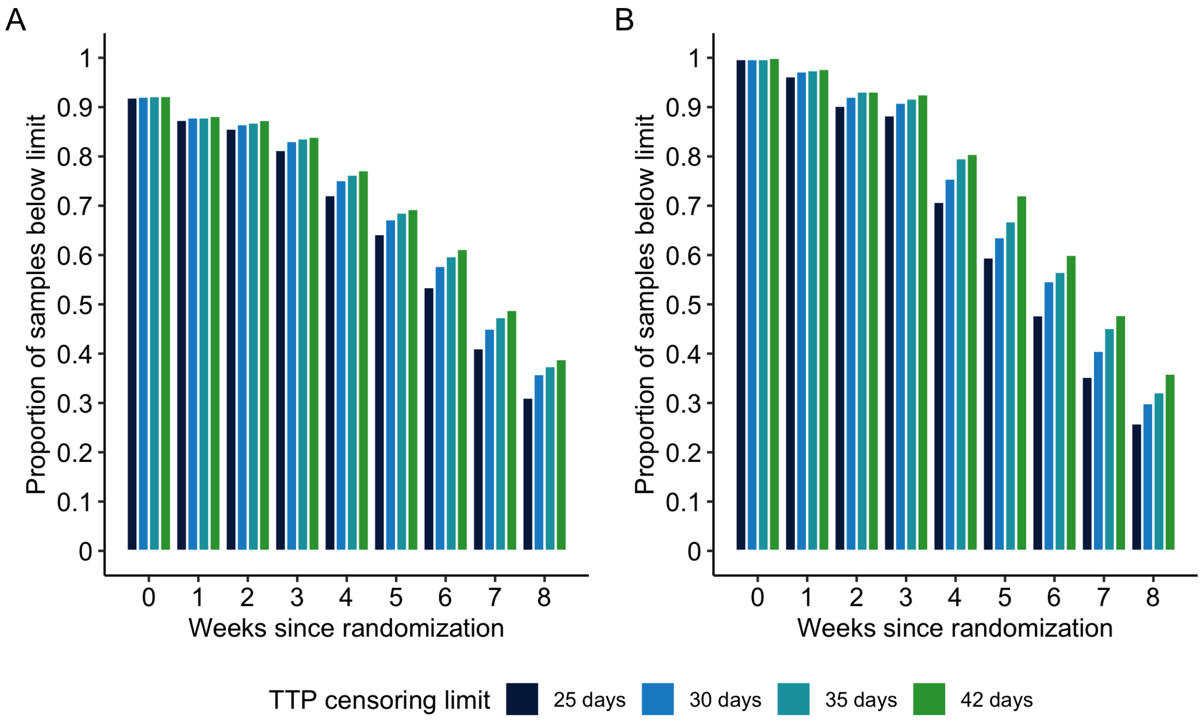

Fig S5. The proportion of samples that return TTP observations below the BACTEC MGIT diagnostic LOD (42 days, green) as well as below various ULOQ$_M$s for each week since randomization for (A) REMox-TB and (B) PanACEA MAMS-TB data.

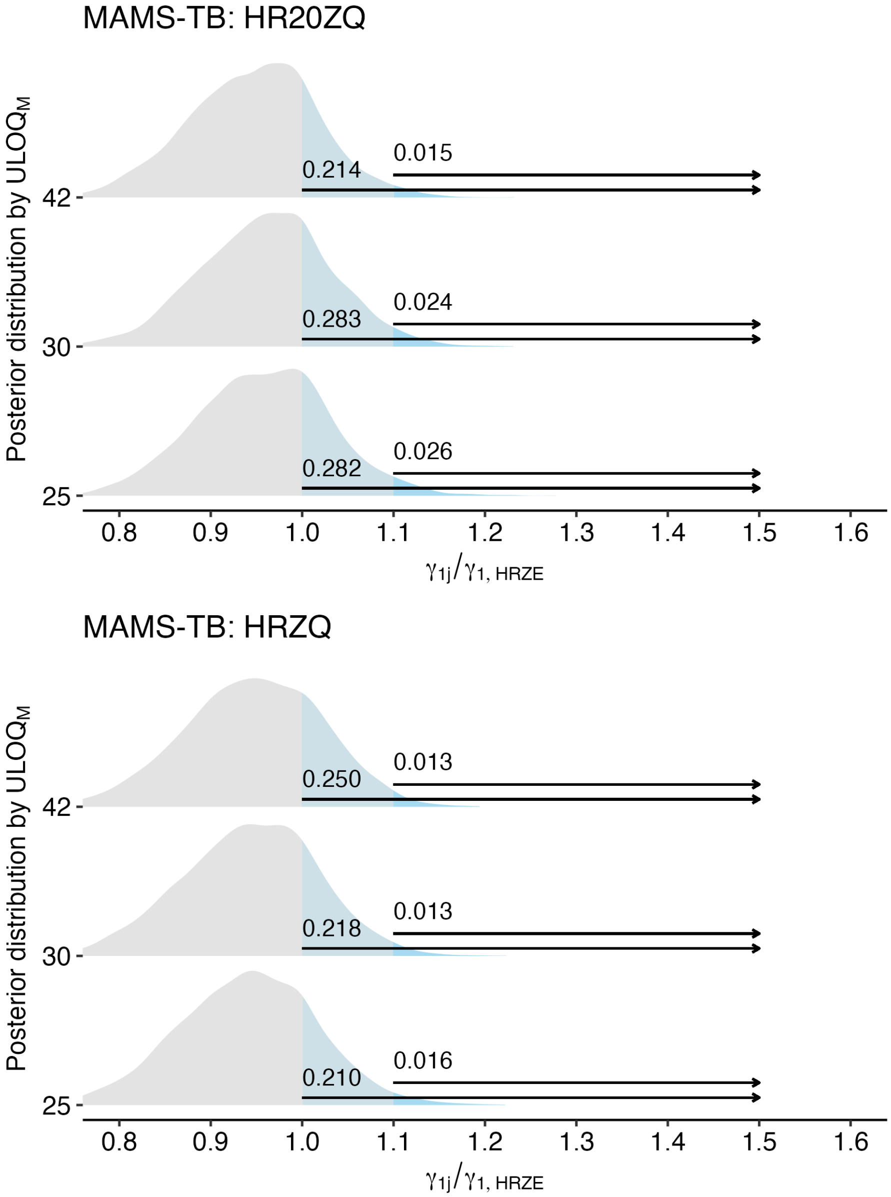

Fig S6. Among regimens with similar or worse bactericidal activity as HRZE, the posterior distributions for the relative comparison of a regimen's slope (γ_{1j}) against the estimated slope on HRZE ($\gamma_{1,\text{HRZE}}$), where a value of 1 indicates equal slopes values (> 1) suggest the regimen has greater bactericidal activity than HRZE. The estimated "confidence" that a regimen has any improvement in bactericidal activity over HRZE ($\Pr(\gamma_{1j}/\gamma_{1,\text{HRZE}} > 1)$) as well as the "confidence" that a regimen has more than 10% improvement in bactericidal activity over HRZE ($\Pr(\gamma_{1j}/\gamma_{1,\text{HRZE}} > 1.1)$) is indicated for each regimen at each ULOQ$_M$. Annotated are the corresponding values for the posterior probabilities of $\Pr(\gamma_{1j}/\gamma_{1,\text{HRZE}} > \tau)$, where τ equals 1 and 1.1, respectively.
